# Deguelin Induces Both Apoptosis and Autophagy in Cultured Head and Neck Squamous Cell Carcinoma Cells

**DOI:** 10.1371/journal.pone.0054736

**Published:** 2013-01-23

**Authors:** Yan-li Yang, Chao Ji, Zhi-gang Bi, Chun-cheng Lu, Rong Wang, Bing Gu, Lei Cheng

**Affiliations:** 1 Department of Otorhinolaryngology, The First Affiliated Hospital, Nanjing Medical University, Nanjing, Jiangsu, China; 2 Department of Dermatology, The First Affiliated Hospital, Nanjing Medical University, Nanjing, Jiangsu, China; 3 Department of Dermatology, BenQ Medical Center, Nanjing Medical University, Nanjing, Jiangsu, China; 4 Key Laboratory of Reproductive Medicine, School of Public Health, Institute of Toxicology, Nanjing Medical University, Nanjing, Jiangsu, China; 5 Laboratory of Reproductive Medicine, The Research Center for Bone and Stem Cells, Nanjing Medical University, Nanjing, Jiangsu, China; 6 Department of Laboratory Medicine, The First Affiliated Hospital, Nanjing Medical University, Nanjing, Jiangsu, China; Complutense University, Spain

## Abstract

Head and neck squamous cell carcinoma (HNSCC) represents more than 5% of all cancers diagnosed annually in United States and around the world. Despite advances in the management of patients with this disease, the survival has not been significantly improved, and the search for potential alternative therapies is encouraging. Here we demonstrate that deguelin administration causes a significant HNSCC cell death. Deguelin induces both cell apoptosis and autophagy by modulating multiple signaling pathways in cultured HNSCC cells. Deguelin inhibits Akt signaling, and down-regulates survivin and cyclin-dependent kinase 4 (Cdk4) expressions, by disrupting their association with heat shock protein-90 (Hsp-90). Deguelin induces ceramide production through de novo synthase pathway to promote HNSCC cell death. Importantly, increased ceramide level activates AMP-activated protein kinase (AMPK), which then directly phosphorylates Ulk1 and eventually leads to cell autophagy. We found that a low dose of deguelin sensitized HNSCC cells to 5-FU. Finally, using a nude mice Hep-2 xenograft model, we also showed a significant anti-tumor ability of deguelin in vivo. Together, we suggest that deguelin may represent a novel and effective chemo-agent against HNSCC.

## Introduction

Head and neck squamous cell carcinoma (HNSCC) represents more than 5% of all cancers diagnosed each year [1]. Standard treatment strategies involve surgery, radiotherapy or chemotherapy [2], [3]. Despite some progresses achieved in these standard strategies recently [4], [5], [6], the survival of HNSCC patients has remained poor [5]. A total of 30–50% of patients develop local or regional recurrence, with more patients develop distant metastases [2], [3]. As such, potential alternative therapies for HNSCC are encouraging.

Deguelin has been recently identified as a potent chemo-preventive agent against a number of cancers. Deguelin suppresses cigarette smoke-induced lung carcinogenesis [7] and the formation of carcinogen-induced aberrant crypt foci in mouse colons [8]. This rotenoid isolated from the African plant *Mundulea sericea* (Leguminosae) has also been shown to inhibit chemically induced skin tumors in mice [9], and preneoplastic lesion in the mouse mammary gland in organotypic culture [10]. In addition, deguelin sensitized leukemia cells to chemotherapeutic agents [11]. The cellular mechanisms of of these effects are not fully studied, though various mechanisms have been proposed, including the suppression of the PI3K/Akt [7], [12] and NF-κB pathways [13], and down-regulation of the apoptosis inhibitor proteins including survivin and XIAP [14]. Recently, deguelin was proposed as an inhibitor of heat shock protein 90 (Hsp 90). Deguelin binds to the ATP binding pocket of Hsp 90 to inhibit its function [15]. Here we study the potential effects of deguelin against HNSCC cells by focusing its mechanisms.

## Materials and Methods

### Chemical and Reagents

3-Methyladenine (3-MA), antibodies against rabbit IgG-HRP, mouse IgG-HRP, tubulin, Bcl-2 and Cdk4 were purchased from Santa Cruz Biotechnology (Santa Cruz, CA). p-AKT (Ser 473), p-AKT (Thr 308), p-S6K (Thr 389), p-AMPKα (Thr 172), p-LKB1 (Ser 428), p-Acetyl-CoA Carboxylase(ACC) (Ser79), p-Ulk1 (Ser317), Ulk1, LC3B (Rabbit mAb), AKT1, cleaved-caspase 3(rabbit mAb), cleaved-caspase 9 (mouse mAb) and Hsp 90 antibodies were purchased from Cell Signaling Technology (Bevery, MA). 5-Aminoimidazole-4-carboxamide ribotide (AICAR) and z-VADfmk were purchased from EMD Bioscience (Shanghai, China). Monoclonal mouse anti-β-actin, tubulin and fumonisin B1 were obtained from Sigma (St. Louis, MO). C6-Ceramide was purchased from Avanti (Alabaster, AB).

### Cell Culture

HNSCC cell lines Hep-2, A253 and SCC-9, pancreatic cell line PANC-1 were maintained in a DMEM medium (Sigma, St. Louis, MO), supplemented with a 10% fetal bovine serum (FBS, Invitrogen, Carlsbad, CA), Penicillin/Streptomycin (1∶100, Sigma, St. Louis, MO) and 4 mM L-glutamine (Sigma, St. Louis, MO), in a CO2 incubator at 37°C.

### Immunoblotting

As reported before [16], [17], aliquots of 30 µg of protein from each sample were separated by 10% SDS–polyacrylamide gel electrophoresis (SDS-PAGE) and transferred onto a polyvinylidene difluoride (PVDF) membrane (Millipore, Bedford, MA). After blocking with 10% instant nonfat dry milk for 1 hour, membranes were incubated with specific antibodies overnight at 4°C followed by incubation with secondary antibodies (HRP-conjugated anti-rabbit or anti-mouse IgG at the appropriate dilutions) for 45 min to 1 hour at room temperature. Antibody binding was detected with the enhanced chemiluminescence (ECL) detection system. Western blots results were quantified using Image J software (downloaded from NIH website) after normalizing to corresponding loading controls.

### Cell Viability Assay (MTT Dye Assay)

Cell viability was measured by the 3-[4,5-dimethylthylthiazol-2-yl]-2,5 diphenyltetrazolium bromide (MTT) method as described early [16], [18].

### Assessment of the Percentage of Apoptotic Cells

As previously reported [16], [18], to detect apoptotic cells, cells were stained with DNA dye Hoechst 33342. Cells with indicated treatments were fixed with 4% formaldehyde in phosphate buffered saline (PBS) for 5 min at 4°C, followed by PBS wash. Cells were then incubated for 20 min with 5 µg/ml of Hoechst 33342 (Sigma, St. Louis, MO) to stain the nuclei. The apoptotic cells were observed under a Confocal Fluorescence microscope. Cells exhibiting condensed chromatin and fragmented nuclei (Hoechst 33342 stain, Blue) were scored as apoptotic cells. For each Hoechst/Calcein experiment, at least 100 cells in 5 random scope fields were counted for apoptotic rate (Magnification 1∶200).

### Enzyme-linked Immunosorbent Assay Cell Apoptosis Detection

As previously reported [16], [18], enzyme-linked immunosorbent assay was used for cell apoptosis detection. The assay was carried out according to the manufacturer’s instructions (Roche Diagnostics, Mannheim, Germany). Briefly, wells of microtitre plates were coated with anti-histone antibody for 120 min. The samples, diluted in incubation buffer, were added (in duplicate) and incubated at room temperature for 120 min. The wells were washed and incubated with anti-DNA-peroxidase solution at room temperature for 90 min. Substrate solution was added and the plates agitated at 250 rpm until the colour developed adequately (approximately 15 min). Measurements were taken at 405 nm and the reference at 490 nm with an automatic microplate analyzer.

### Caspase Activity

Caspase 3 activity was determined using Caspase-Glo-3 assays from Promega. This assay provides luminogenic substrate in a buffer system optimized for each specific caspase activity. The caspase cleavage of the substrate is followed by generation of a luminescent signal. The signal generated is proportional to the amount of caspase activity present in the sample. Protein (10 µg) from the cell samples was diluted in water to a final volume of 50 µL and added to a white 96-well microtitre plate, followed by 50 µl of Caspase-Glo-3 reagent. The plate was sealed and gently mixed at 300–500 rpm for 30 s and incubated at room temperature for 30 min. Luminescence was measured in a microplate reader (TECAN Infinite 200).

### Immunoprecipitation (IP)

As previously reported [18], Hep-2 cells with indicated treatments were lysed with lysis buffer (200 mM NaCl (pH 7.4), 1% Triton X-100, 10% glycerol, 0.3 mM EDTA, 0.2 mM Na_3_VO_4_, and protease inhibitor cocktails (Roche Diagnostics, Indianapolis, IN). Aliquots of 850 µg of proteins from each sample were precleared by incubation with 15 µl of protein A/G Sepharose (beads) (Amersham, IL) for 1 hour at 4°C. Precleared samples were incubated with specific antibodies (1 µg/sample) in lysis buffer (850 µl) overnight at 4°C. To this was added 30 µl of protein A/G beads and the samples were incubated for 2 hours at 4°C. The beads were washed five times with phosphate-buffered saline (PBS) and once with lysis buffer, boiled, separated by 10% SDS-PAGE, and transferred onto a PVDF membrane followed by Western blotting analysis as described above.

### Analysis of Cellular Ceramide Level

As previously reported [18], [19], [20], the total pool of sphingolipids in Hep-2 cells were radio-labeled by growing the cells in the presence of 3 µCi/ml[3H]l-serine (30 Ci/mmol; Amersham), a precursor for sphingolipid biosynthesis. After indicated treatment, the medium was removed, and the cells were fixed in ice-cold CH3OH, followed by lipid extraction from the cells [21]. Aliquots of the lipid extracts were taken for the determination of the total amount of lipid-incorporated radioactivity. Acylglycerolipids were hydrolyzed during 1 h of incubation at 37°C in CHCl3/CH3OH (1∶1, v/v) containing 0.1 m KOH. The remaining lipids were re-extracted and applied on high performance thin-layer chromatography plates. Plates were developed in CHCl3/CH3OH/H2O (14∶6:1, v/v) in the first dimension and in CHCl3/CH3COOH (9∶1, v/v) in the second dimension to resolve Ceramide. Ceramide-containing spots were scraped and subjected to scintillation counting. The effects of TSA and inhibitors on Ceramides levels were determined by growing the cells, after overnight adherence, for 4 days in medium containing [^3^H]serine. Drugs were present for the last 2-day period or the final 12 h before the end of incubation. Treatment with 0.1 units/ml bacterial sphingomyelinase (Sigma) for the final 2 h of the incubation served as positive control (maximal Ceramide formation from sphingomyelin; see Ref. [21]. Cellular Ceramide production was indicated as fold change compared to untreated control.

### AMPKα1siRNA Knockdown

AMPKα1-specific SMARTpool siRNA reagents (M-005027) were purchased from Millipore (Schwalbach, Germany).An non-silencing scramble siRNA was obtained from Santa Cruz Biotechnology (Santa Cruz, CA). Hep-2 cells were seeded in a 6-well plate 2 day prior to transfection and cultured to 60% confluence the following day in 1% FBS medium. For RNAi transfection, 3.2 µl PLUS™ Reagent (Invitrogen, Carlsbad, CA) was diluted in 90 µl of RNA dilution water (Santa Cruz, CA) for 5 min in room temperature. Then, 100 nM of siRNA (AMPKα1 and scramble) was added to PLUS™ Reagent and left for 5 min at room temperature. To this was added 3.6 µl of Lipofectamine (Invitrogen, Carlsbad, CA) and incubation for another 30 mins. Finally, the complex was added to the wells containing 1.5 ml of medium, cells then cultured for another 48 hours, transfection efficiency was monitored by co-transfected green fluorescence protein (GFP, 0.05 µg/well) under a fluorescence scope, at least 50% of cells were GFP positive before further each experiments. Western blots were also performed to test expression level of AMPKα1 to further confirm transfection efficiency.

### In vivo Nude Mice Hep-2 Xenograft Assay

Female BALB/c nude mice, 8–10 weeks of age, weighing 22 to 34 g, were acclimatized for 1 week before being injected s.c. with 3.5×10^6^ Hep-2 cells that had been re-suspended in 100 µL of medium. After 5 days when established tumors around 0.3 cm^3^ in diameter were detected, mice were randomized and divide in two groups. Ten mice per group were orally treated with deguelin (4 mg/kg) on days 1, 3, and 5 of each week for 3 weeks. Control animals received equal volume of saline solution. Tumor size were measured weekly by the modified ellipsoid formula: (π/6)×AB^2^, where A is the longest and B is the shortest perpendicular axis of an assumed ellipsoid corresponding to tumor mass [22], [23].

### Statistical Analysis

Individual culture dishes or wells were analyzed separately (no pooling of samples was used). In each experiment a minimum of three wells of each treatment was used. Each experiment was repeated a minimum of three times. In each experiment, the mean value of the repetitions was calculated and this value was used in the statistical analysis. All data were normalized to control values of each assay and are presented as mean ± SEM. Data were analyzed by one-way anova followed by a Scheffe’s f-test by using SPSS software (SPSS Inc., Chicago, IL, USA). Significance was chosen as *P*<0.01.

## Results

### Cytotoxic Effects of Deguelin in Cultured HNSCC Cells

**Figure 1:**
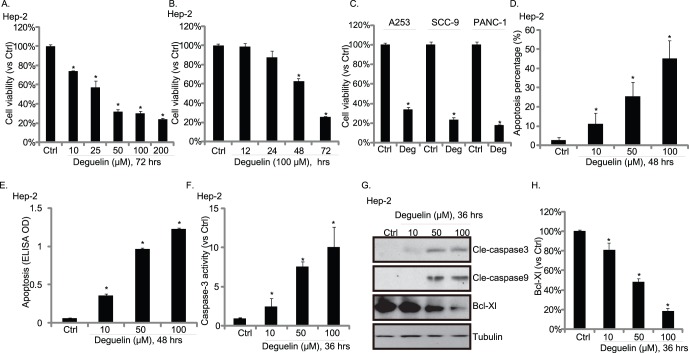
Cytotoxic effects of deguelin in cultured HNSCC cells. Hep-2 cells were exposed to deguelin at a various concentration (10, 25, 50, 100 and 200 µM) for 72 hours (A) or exposed to 100 µM of deguelin for different time points (12, 24, 48 and 72 hours) (B), cell viability was then measured by MTT assay. (C) Three different cell lines A253, SCC-9 and PANC-1 were exposed to 100 µM of deguelin (Deg) for 72 hours; cell viability was measured by MTT assay. Hep-2 cells were treated with indicated concentration of deguelin, hoechst nuclear staining and enzyme-linked immunosorbent cell apoptosis assay were utilized to analyze cell apoptosis (D–E), caspase-3 activity was also measured (F), apoptosis related proteins including cleaved caspase-3, cleaved caspase-9, Bcl-Xl were detected by Western blots (G), Bcl-Xl expression level was quantified using image J software (H). The mean of at least three independent experiments performed in triplicate is shown. Statistical significance was analyzed by ANOVA. *p<0.01 vs Control.

In cultured Hep-2 cells, deguelin (see structure in Fig. S1A) administration caused significant cell viability loss (growth inhibition) in both dose- (Fig. 1A) and time- dependent (Fig. 1B) manner. Similar results were also observed in two other HNSCC cell lines A253 and SCC-9, and in pancreatic cancer cell line PANC-1 (Fig. 1C). Hoechst nuclear staining and enzyme-linked cell apoptosis assay were used to test cell apoptosis in deguelin-treated cells, a significant cell apoptosis was observed in deguelin-treated Hep-2 cells (Fig. 1D and E). Further, deguelin exposure increased caspase-3 activity (Fig. 1F), caused caspase-3 and caspase-9 cleavage (Fig. 1G), while down-regulating Bcl-xl expression (Fig. 1G–H).

### Deguelin Down-regulates Akt Signaling

**Figure 2:**
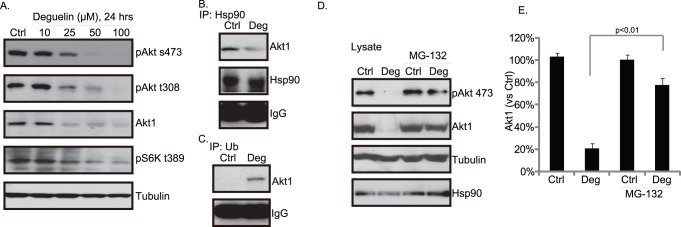
Deguelin down-regulates Akt signaling. Hep-2 cells were exposed to deguelin at indicated concentration (10, 25, 50 and 100 µM) for 24 hours, total- and phospho- levels of Akt as well as phosphorylation level of S6K were measured by Western blot using indicated antibodies (A).The association between Akt and Hsp 90 and the association between Akt and ubiquitin after deguelin treatment (Deg, 100 µM, 12 hours) were measured by immunoprecipitation (IP) assay (B-C). Effect of MG-132 (1 µM, 12 hour pretreatment) on deguelin-induced Akt degradation was shown in (D–E). The mean of at least three independent experiments performed in triplicate is shown. Statistical significance was analyzed by ANOVA.

Published studies demonstrated that deguelin inhibits Akt and downstream mammalian target or rapamycin (mTOR) signaling [7], [12], considering that activation of Akt contributes to HNSCC progression [24], we then examined deguelin’s role on Akt signaling. Western blots results in Fig. 2A demonstrated that deguelin down-regulated the expression and phosphorylation of Akt in Hep-2 cells, S6K phosphorylation (Akt downstream signal) was also inhibited. Immunoprecipitation (IP) assay results showed that Akt associated with Hsp 90 in control Hep-2 cells, and deguelin disrupted it (Fig. 2C), which could lead to Akt degradation by its association with ubiquitin (Fig. 2C). As a matter of fact, MG-132, an ubiquitin dependent degradation pathway inhibitor, almost reversed deguelin-induced Akt degradation (Fig. 2D–E). These data suggest that deguelin down-regulates Akt signaling probably by disrupting its association with Hsp 90 in cultured HNSCC cells.

### Deguelin Down-regulates Survivin and Cdk4

**Figure 3:**
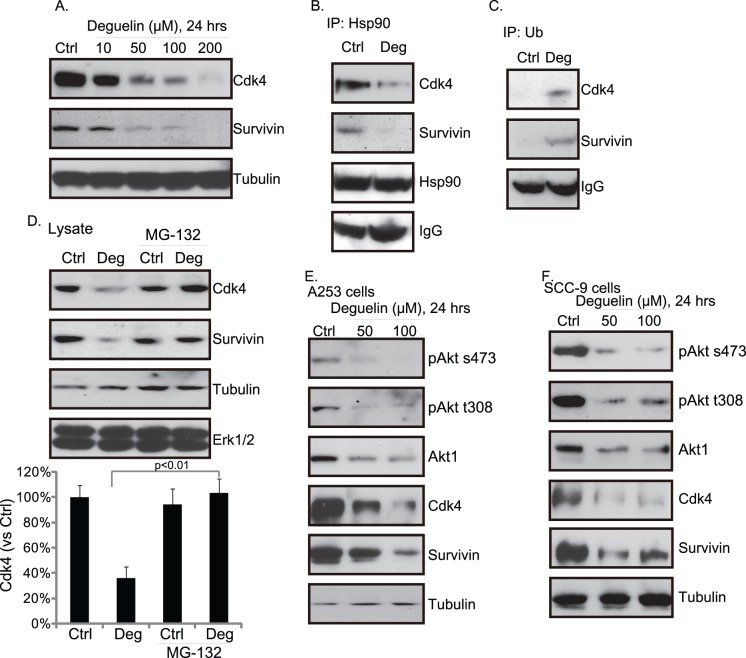
Deguelin down-regulates survivin and Cdk4. Hep-2 cells were exposed to deguelin at indicated concentration (10, 25, 50 and 100 µM) for 24 hours, expression levels of survivin, Cdk4 and tubulin were measured by western blot (A).The association between survivin, Cdk4 and Hsp 90 as well as the association between survivin, Cdk4 and ubiquitin after deguelin treatment (Deg, 100 µM, 12 hours) were detected by immunoprecipitation (IP) assay (B–C). Effect of MG-132 (1 µM, 12 hour pretreatment) on deguelin-induced Cdk4 and survivin degradation was shown in (D). Western blots was performed to test the expression of Akt, phos-Akt, survivin, Cdk4 and tubulin after deguelin treatment in A253 (E) and SCC-9 cells (F). The mean of at least three independent experiments performed in triplicate is shown. Statistical significance was analyzed by ANOVA.

**Figure 4:**
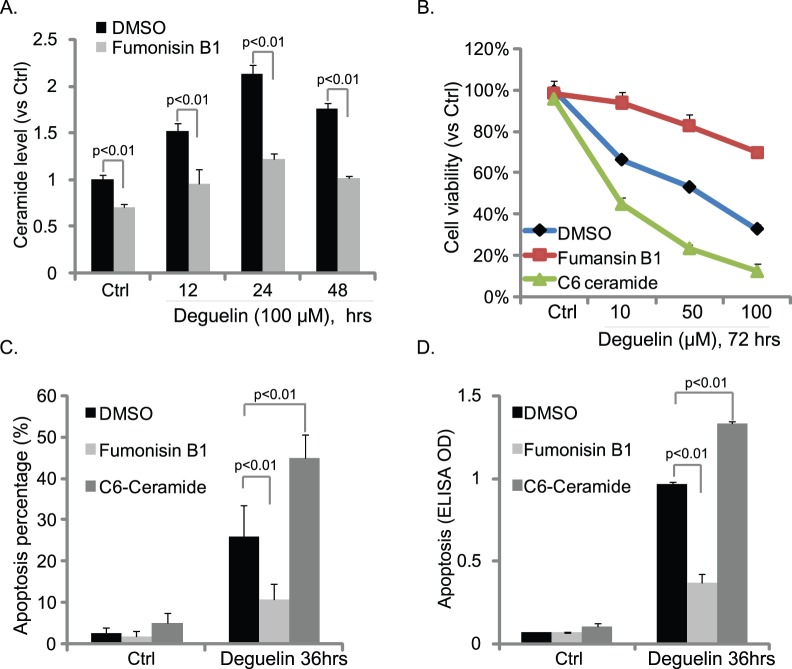
Deguelin induces cellular ceramide synthesis. Hep-2 cells were exposed to 100 µM of deguelin with or without fumonisin B1 (20 µM) for indicated time points, intracellular ceramide level was analyzed using methods mentioned above (A). Hep-2 cells were treated with fumonisin B1 (20 µM) or C6-ceramide (10 µg/ml), followed by deguelin (100 µM) exposure, MTT assay was used to test cell viability after 72 hours (B), Hoechst staining (C) and enzyme-linked immunosorbent cell apoptosis assay (D) were utilized to test Hep-2 cell apoptosis after 48 hours. The mean of at least three independent experiments performed in triplicate is shown. Statistical significance was analyzed by ANOVA.

As a ubiquitous molecular chaperone, Hsp 90 induces the conformational maturation and stabilization of numerous client proteins including Akt [25], survivin [26] and Cyclin-dependent kinase 4 (Cdk4) [27], [28]. On the other hand, inhibition of Hsp 90 prevents the stabilization of these client proteins, eventually resulting in their degradation [29]. Recently, studies have proposed that deguelin is a novel inhibitor of Hsp 90 [15], [29]. In figure 2, we have demonstrated that deguelin caused Akt degradation, so we next dictated the expression levels of survivin and Cdk4 after deguelin exposure in Hep-2 cells. Western blot results in Fig. 3A showed that deguelin exposure dose-dependently down-regulated the expression of survivin and Cdk4 in Hep-2 cells. Further, IP experiments in Fig. 3B–C demonstrated that deguelin disrupted the association between Hsp 90 with survivin and Cdk4 (Fig. 3B), while promoting the association between ubiquitin with survivin and Cdk4 (Fig. 3C). Again, MG-132 reversed deguelin’s effect on survivin and Cdk4 expression (Fig. 3D). Results in Fig. 4E–F illustrated the similar effect of deguelin on Akt/cyclin/Cdk4 in two other HNSCC lines (A253 and SCC-9).

### Deguelin Induces Cellular Ceramide Synthesis

As plasma membrane sphingolipids, ceramides are biologically active signaling molecules with numerous regulatory effects on key cellular functions. Ceramides are known apoptosis intermediate molecules [30]. Studies including ours [16], [18], [19], [20] have shown the very interesting relationship between ceramides and apoptosis in cancer cells. The processes that enhance intracellular ceramides accumulation, either by enhanced synthesis or reduced metabolic removal, would provide favorable pro-apoptotic outcomes [16], [18], [31], [32]. As such, we tested the cellular ceramide levels after deguelin exposure in Hep-2 cells. As shown in Fig. 4A, ceramide level increased after deguelin exposure by almost two folds in Hep-2 cells. The ceramide *de novo* synthase inhibitor fumonisin B1 [33], [34] almost blocked deguelin-induced ceramide production (Fig. 4A). Deguelin-induced cell death (Fig. 4B) and apoptosis (Fig. 4C–D) were also inhibited by fumonisin B1. Reversely, exogenously adding cell permeable short chain C6 ceramide dramatically enhanced cell viability loss and apoptosis by deguelin (Fig. 4B–D). These results suggested that deguelin treatment increases cellular ceramide level through *de novo* synthase pathway to mediate HNSCC cell death and apoptosis.

### Deguelin Activates AMPK Signaling

**Figure 5:**
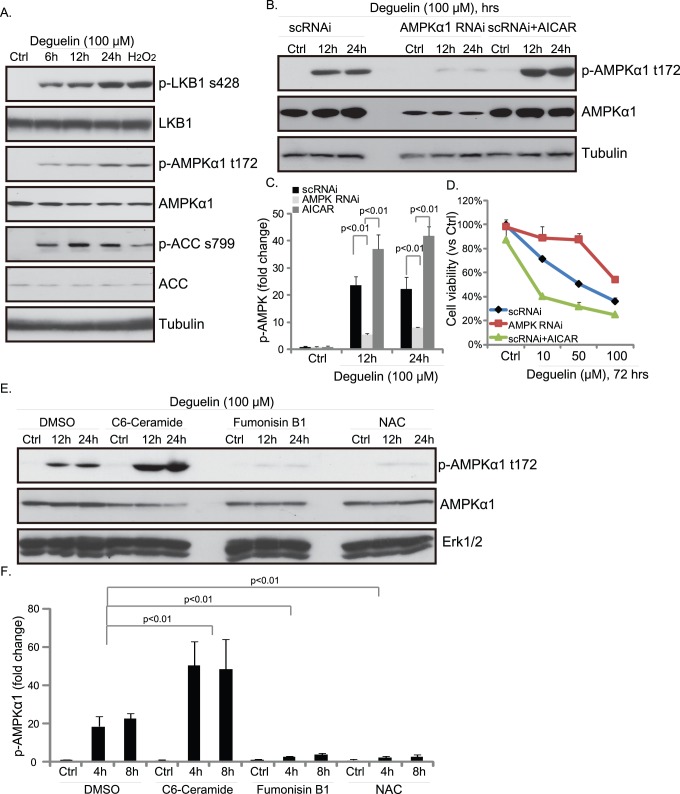
Deguelin activates AMPK signaling. Hep-2 cells were exposed to 100 µM of deguelin for indicated time points, followed by western blot detecting phospho- and total- level of LKB1-AMPK-ACC using specific antibodies (A). Hep-2 cells were transfected with scramble or AMPKα1RNAi (100 nM each) for 48 hours. Western blot was utilized to test AMPKα1 expression after transfection. Successfully AMPK knockdown cells and their parental cells were treated with deguelin (100 µM) in the presence or absence of AICAR (1 mM), phospho- and total- level of AMPKα1 were analyzed in (B–C), MTT assay was then used to test cell viability after 48 hours (D). Hep-2 cells were pretreated with fumonisin B1 (20 µM), C6-ceramide (10 µg/ml) or NAC (500 µM) for 1 hr before deguelin exposure for indicated time points, phospho- and total level of AMPKα1 as well as tubulin were tested by western blots (E–F). The mean of at least three independent experiments performed in triplicate is shown. Statistical significance was analyzed by ANOVA.

Our previous study shows that doxorubicin induces ceramide production to activate AMP-activated protein kinase (AMPK), which mediates cell apoptosis [16]. Since deguelin increases cellular ceramide level in Hep-2 cells (See Fig. 4), we then tested deguelin’s effect on AMPK activation. Western blot results in Fig. 5A demonstrated that deguelin induced a significant AMPK activation as LKB1-AMPK-ACC phosphorylation went up after deguelin treatment in Hep-2 cells. Positive control H_2_O_2_ also induced significant AMPK activation (Fig. 5B). RNAi knocking-down AMPKα1 inhibited AMPKα1 phosphorylation and cell death (viability loss) induced by deguelin, reversely, AMPK activator AICAR enhanced deguelin-induced Hep-2 cell death (Fig. 5B–D). To test the possible involvement of ceramide in deguelin-induced AMPK activation, we again utilized short chain cell permeable C6-ceramide (positive) and ceramide de novo synthase inhibitor fumonisin B1 (negative), results in Fig. 5E–F showed that inhibition of ceramide production by fumonisin B1 largely inhibited AMPK activation by deguelin, while C6-ceramide enhanced it. Interestingly, anti-oxidant n-acetyl-cysteine (NAC) also diminished deguelin-induced AMPK activation. These results suggested that ceramide mediated AMPK activation is important for Hep-2 cell death induced by deguelin.

### AMPK Dependent Autophagy Pathway Contributes to Deguelin Induced Hep-2 Cell Death

**Figure 6:**
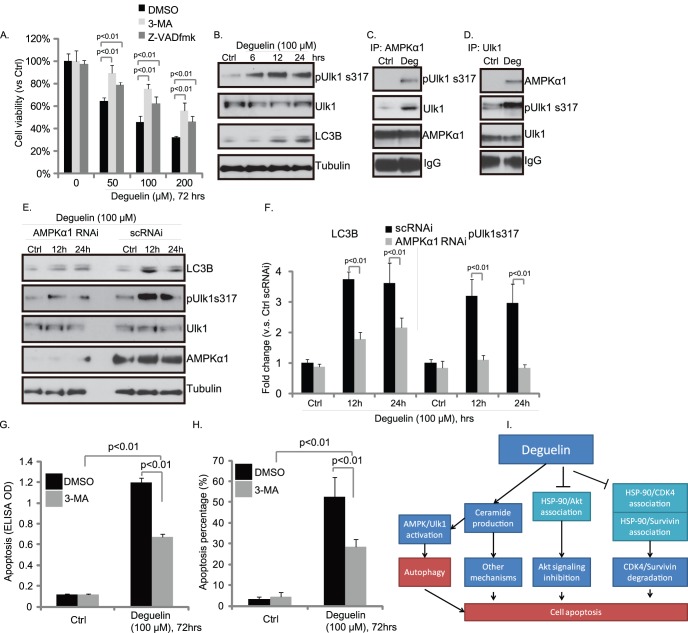
AMPK-dependent autophagy pathway contributes to deguelin-induced Hep-2 cell death. Hep-2 cells were pretreated with Z-VAD-fmk (50 µM) or 3-MA (2 mM) for 1 hr before deguelin exposure for 72 hours, cell viability was measured by MTT assay (A). Hep-2 cells were exposed to 100 µM of deguelin for indicated time points, followed by western blot detecting LC3B, tubulin, phospho- and total- level of Ulk1 (B). The association between AMPKα1 and Ulk1 after deguelin exposure (100 µM, 12 h) was determined by IP assay (C–D). Hep-2 cells were transfected with scramble or AMPKα1 siRNA (100 nM) for 48 hours. Western blot was then utilized to test AMPKα expression in transfected cells. Successfully AMPK knocking-down cells and their parental cells were treated with deguelin for indicated time points. AMPK, LC3B, tubulin, phospho- and total- level of Ulk1 were tested by western blots (E), LC3B and phospho-Ulk1 levels were quantified (F). Hep-2 cells were pretreated with 3-MA (2 mM) for 1 hr before deguelin (100 µM) exposure for 72 hours, cell apoptosis was measured by enzyme-linked immunosorbent cell apoptosis assay (G) and hoechst nuclear staining (H). (I) The proposed signaling pathway in this study: deguelin induces both cell apoptosis and autophagy by modulating multiple signaling pathways in cultured HNSCC cells: deguelin inhibits Akt signaling, and down-regulates survivin and cyclin-dependent kinase 4 (Cdk4) expressions, by disrupting their association with Hsp-90. Deguelin induces ceramide production through *de novo* synthase pathway to promote HNSCC cell death. Ceramide activates AMPK, which directly phosphorylates Ulk1 to promote an early cell autophagy. Cell autophagy is pro-apoptotic in our system. The mean of at least three independent experiments performed in triplicate is shown. Statistical significance was analyzed by ANOVA.

MTT results in Fig. 4A demonstrated that general caspase inhibitors z-VADfmk reduced, but not reversed, deguelin induced Hep-2 cell death, suggesting that other cell death pathways might also be involved. As a matter of fact, autophagy inhibitor 3-methyladenine **(**3-MA) reduced deguelin-induced Hep-2 cell death (viability loss) to suggest that autophagy may be involved (Fig. 6A). Importantly, we discovered that deguelin induced Ulk1 phosphorylation at serine 317 (AMPK phosphorylation site [35]) and its association with AMPK (Fig. 6C–D). Deguelin also induced LC3B up-regulation, a key upstream indicator of autophagy [35], [36]. Further, AMPK RNAi reduced deguelin-induced Ulk1 phosphorylation and LC3B up-regulation in Hep-2 cells (Fig. 6E–F). These data suggested that AMPK/Ulk1 dependent autophagy pathway may also contribute to deguelin-induced cytotoxicity effect. We found that Ulk1 phosphorylation and LC3B up-regulation (indicators of autophagy) were induced 12–24 hours after deguelin treatment (Fig. 6B), while no significant apoptosis was observed at that time point (Fig.S1B–C). The apoptosis ELISA OD and hoechst percentage were comparable to the untreated control cells at 24 hours time point (Fig. S1B and C) to suggest that apoptosis was induced later after autophagy. More importantly, autophagy inhibitor 3-MA reduced deguelin-induced Hep-2 cell apoptosis (Fig. 6G and H). These data suggest that deguelin-induced autophagy is pro-apoptotic at least in our system. However, it should be noted that 3-MA didn’t totally abolished cell apoptosis by deguelin (Fig. 6G–H), suggesting that apoptosis induction by deguelin is not solely dependent on autophagy (See Fig. 6I), Based on our available data, we conclude that autophagy is an early event after deguelin treatment in Hep-2 cells and is pro-apoptotic, and multiple signaling events together contribute to cell death and Hep-2 cell apoptosis by deguelin (Fig. 6I).

### Low Dose of Deguelin Sensitizes Hep-2 Cells to 5-FU

**Figure 7:**
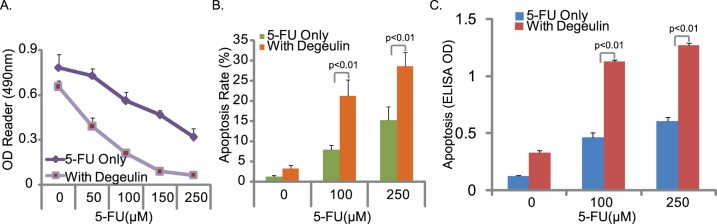
Low dose of deguelin sensitizes Hep-2 cells to 5-FU. Hep-2 cells were exposure with the indicated concentration of 5-FU in the presence or absence of deguelin (10 µM), MTT assay was used to test cell viability after 72 hours (A), Hoechst 33342 nuclear staining (B) and enzyme-linked immunosorbent cell apoptosis assay (C) were used to test cell apoptosis after 48 hours. The mean of at least three independent experiments performed in triplicate is shown. Statistical significance was analyzed by ANOVA.

5-fluorouracil (5-FU) is widely used in the treatment of HNSCC. However, development of drug resistance is one of the major causes of HNSCC treatment failure. The goal of this next set of experiments was to test the effect deguelin on 5-FU sensitivity in Hep-2 cells. Results in Fig. 7 demonstrated that a relative low dose of deguelin (10 µM) dramatically enhanced 5-FU-induced Hep-2 cell death (Fig. 7A) and apoptosis (Fig. 7B–C). These results confirmed that deguelin sensitizes *Hep-2* cells to 5-FU.

### Deguelin’s in vivo Anti-tumor Effects in a Hep-2 Xenograft Model

**Figure 8:**
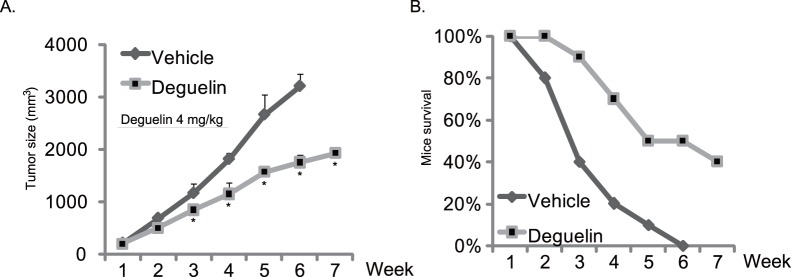
Deguelin’s *in vivo* anti-tumor effects in a Hep-2 xenograft model. Female BALB/c nude mice, 8–10 weeks of age, weighing 22 to 34 g, were acclimatized for 1 week before being injected s.c. with 3.5×10^6^ Hep-2 cells that had been re-suspended in 100 µL of medium. After 5 days when established tumors around 0.3 cm^3^ in diameter were detected, mice were randomized and divide in two groups. Ten mice per group were orally treated with deguelin (4 mg/kg) on days 1, 3, and 5 of each week for 3 weeks. Control animals received equal volume of saline solution. Tumor size was measured weekly using method mentioned above (A). Mice survival rate were also measured in (B). *p<0.05* vs. vehicle control group. Statistical significance was analyzed by ANOVA.

Finally, we tested the *in vivo* anti-tumor effect of deguelin in a Hep-2 xenograft model as described in material and methods. The Hep-2 xenograft mice group that received indicated oral deguelin treatment showed a great inhibition in tumor growth, which is demonstrated by reduced tumor size (Fig. 8A) and improved mice survival and (Fig. 8 B), indicating a significant anti-tumor ability by deguelin *in vivo.*

## Discussion

HNSCC refers to a large heterogeneous group of tumors, which include the face, nasopharynx, oral cavity, oropharynx, hypopharynx, and/or larynx. The worldwide incidence exceeds half a million cases annually [37]. The most prevalent histologic type of head and neck cancer is squamous cell carcinoma, which represents >90% of cases diagnosed each year [38]. Despite multiple significant progresses have been made in treatments including surgery, radiation, and chemotherapy in recent years, HNSCC continue to have the one of the worst 5-year survival rates in all cancers [3], [37], [39]. Current standard chemotherapy options for HNSCC patients involve the use the chemotherapy drugs cisplatin and 5-fluorouracil (5-FU). Radiation is delivered either following or with chemotherapy. Each of these treatment options has multiple associated toxicities, including xerostomia, dysphagia, fever, leukopenia, anorexia, and difficulty in assessing recurrence [40]. As such, searching novel therapeutic agents/adjuvants is necessary and urgent.

The molecular chaperone Hsp 90 participates in preserving the expression and activity of various proteins that are critical for the progression of cancers and ensure unlimited growth of tumors and their resistance to chemotherapy and radiotherapy. These proteins including hypoxia-inducible factor 1α (HIF-1α) [41], [42], p53 [43], [44], Akt [25], survivin [26], Cyclin-dependent kinase 4 (Cdk4) [27], [28] and many others. Upon inhibition of the Hsp 90 chaperone function, such client proteins are destabilized and degraded in a ubiquitin-dependent manner, which disrupts multiple pathways essential for tumor cell progression; hence, pharmacological Hsp 90 inhibitors could be an useful anticancer agents [29]. As a matter of fact, several Hsp 90-inhibiting compounds are currently tested in preclinical or phase I–III clinical trials as single anti-cancer agents or in combination with conventional drugs and radiation [29]. One of the recent discovered Hsp 90 inhibitors is deguelin [15], [29], here we found that deguelin depleted Hsp 90 client proteins including Akt, survivin and Cdk4 by disrupting the their association with Hsp 90, all these proteins are proven to be important for cancer development for HNSCC [12], [45], [46].

As well as the numerous metabolic targets of AMPK for which it is best known, it is becoming increasingly clear that AMPK has many other downstream signaling effectors. Recent findings including ours [16] have suggested that sustained AMPK activation inhibits cell growth and proliferation, and also induces cell death and apoptosis (45–54). Given that cell growth, DNA replication, and mitosis are all major consumers of ATP, and also that the upstream regulator of AMPK, LKB1, is known to be a tumor suppressor, these findings are not surprising. Our previous study suggests that ceramide production mediates AMPK activation in doxorubicin treated cancer cells, which then leads to cancer cell apoptosis [16]. Consistent with these findings, we discovered here that deguelin also caused ceramide production through *de novo* synthesis pathway to mediate AMPK activation and HNSCC cell death.

Autophagy is a catabolic process involving the degradation of a cell’s own components through the lysosomal machinery. It is a tightly-regulated process that plays a critical role in cell growth, development, and death. Though basal or low level of autophagy is cell-protective, excessive or sustained autophagy cause cancer cell death [47]. As a key energy sensor and regulates cellular metabolism to maintain energy homeostasis, activation of AMPK promotes autophagy by directly activating Ulk1 through direct phosphorylation of Ser 317 and Ser 777 [48], [49]. Conversely, autophagy is inhibited by the mammalian target of rapamycin (mTOR), the latter a central cell-growth regulator that integrates growth factor and nutrient signals, inhibition of mTOR signaling promotes autophagy [50]. Here we found that AMPK activation by deguelin directly phosphorylates Ulk1 at ser 317 to mediate cell autophagy, which also contributes to HNSCC cell death.

A very recent study by Yao et al., shows that perifosine induces cell apoptosis in human osteosarcoma cells, perifosine blocks Akt/mTOR complex 1 (mTORC1) signaling and inhibits survivin expression by disrupting their association with heat shock protein-90 (HSP-90) [51]. Perifosine is also shown to induce pro-apoptotic ceramide production and AMPK activation [20], [52], [53]. These effects are quite similar to what we reported here in deguelin-treated Hep-2 cells. Further, our groups [16], [54] and others have demonstrated that multiple anti-cancer chemotherapies including vincristine [55], [56], taxol [52], [57], temozolomide [58], and doxorubicin [16], [59], as well as natural anti-cancer plant extracts including ursolic acid [60], Honokiol [61], widdrol [62], EGCG [63], quercetin [64] and fisetin [65] all activate AMPK to promote cancer cell death. A very recent study by Din et al., show that activation of AMPK by aspirin inhibits mTOR activation and induces autophagy cell death in colorectal cancer cells both in vivo and in vitro [66]. Further, Zhou’s group recently shows that AMPK activator AA005 induces mTORC1 inhibition and autophagy cell death in colon cancer cells [67]. These latest results are also similar to our results presented here.

In the current study, we also showed a significant *in vivo* anti-tumor ability of deguelin in a nude mice xenograft model. The mice group that received oral deguelin administration showed improved mice survival and reduced tumor size. In the follow-up study, we will also test if the combination of deguelin with 5-FU or other chemo-drugs will achieve a more significant anti-tumor effect *in vivo*. We will also examine if the *in vivo* anti-Hep-2 ability of deguelin (with or without 5-FU) is associated with its effects on the signaling pathways as we observed in the *in vitro* studies.

### Conclusions

Here, we demonstrated that deguelin promoted HNSCC cell death by inducing both cell apoptosis and autophagy *in vitro*. Acting as an Hsp 90 inhibitor, deguelin caused Akt, survivin and Cdk4 degradation by disrupting their association with Hsp 90. Deguelin also induced pro-apoptotic ceramide accumulation. Meanwhile, increased ceramide activated LKB1/AMPK/Ulk1 signaling pathway to promote cell autophagy. It is also demonstrated a low dose of deguelin sensitized HNSCC cells to 5-FU. We suggest that deguelin may represent as a novel and effective agent against HNSCC.

## Supporting Information

Figure S1 (A) The structure of deguelin. Hep-2 cells were exposed to 100 µM of deguelin for indicated time points, cell apoptosis was examined by enzyme-linked by enzyme-linked immunosorbent cell apoptosis assay (B) and hoechst nuclear staining (C). Note that there was no apoptosis induction at 24 hours after deguelin treatment. The mean of at least three independent experiments performed in triplicate is shown. Statistical significance was analyzed by ANOVA. * *p*<0.01 vs Control.
(EPS)
